# Illustrations of the Efficacy of Compression and Percussion in the Cure of Rheumatism and Sprains, Scrofulous Affections of the Joints and Spine, Chronic Pains Arising from a Scrofulous Taint in the Constitution, Lameness, and Loss of Power in the Hands from Gout, Paralytic Debility of the Extremities, General Derangement of the Nervous System; and in Promoting Digestion, with All the Secretions and Excretions

**Published:** 1824-08

**Authors:** William Balfour

**Affiliations:** the University of Edinburgh.


ART. II.—*Illustrations of the Efficacy of Compression and Percussion in the Cure of Rheumatism and Sprains, Scrofulous Affections of the Joints and Spine, chronic Pains arising from a Scrofulous Taint in the Constitution, Lameness, and Loss of Power in the Hands from Gout, Paralytic Debility of the Extremities, General Derangement of the Nervous System; and in promoting Digestion, with all the Secretions and Excretions.* By WILLIAM BALFOUR, M.D. of the University of Edinburgh.

[Continued from vol. li. page 462.]

CASE XXIV.—A lady, aged sixty-one, unmarried, consulted me on the 21st February, 1822, for rheumatism, of three years' standing, in her loins, left hip-joint, and whole of the thigh, which rendered her quite lame. The motion of the joint was so impaired, that she could not advance the left foot before the right, and, therefore, when she walked, it was by putting forward the right, and bringing up the left. Rotation of the thigh was also very obscure and painful, and a grating in the interior of the joint showed the articular surfaces were dry. From pain and weakness in the left loin, the patient leaned forward, and inclined manifestly to that side. The pain extended, anteriorly, to the crest of the ilium, top of the haunch-bone, downwards and forwards into the groin; posteriorly, downwards behind the trochanter major to the tuber ischii (seat-bone). There was great pain also all round the neck of the thigh-bone, the base of the trochanter major, in the front of the joint exterior to the crural nerve and artery, and in the inside of the thigh, at the insertion of the adductor muscles. These were the most painful points, though the whole thigh, particularly the inside, was affected to the very knee.

This lady, from a false sense of delicacy, entertained an almost insuperable aversion to my mode of treatment. But it was the dernier resort—her only resource: all other means had failed, and her complaints were daily gaining ground. She was compelled, therefore, to submit; and, when she saw there was nothing in my mode of proceeding that could occasion a blush in a girl of sixteen, she regretted exceedingly she had been so long the victim of her groundless apprehensions.

Pain was soon removed from the tuber ischii, the base of the trochanter major, and crest of the ilium; but not so easily from the loins, hipjoint, and insertion of the adductor muscles. By compression and percussion, however, alternated with passive motion of the joint, the affection of these parts was so far subdued in a month, that the patient could walk perfectly. Some of her acquaintance, whom she kept in ignorance of her making any further efforts to obtain a cure, congratulated her upon the improvement they observed in her walk and gait, remarking "she did not seem to limp at all now, and was as erect as ever." The operations were continued occasionally a month longer, when pain was completely removed from every part, and the use of the hip-joint perfectly restored.

In my preliminary observations, I have remarked that compression and percussion have a salutary influence on digestion. The observation was remarkably verified in this lady's case. After having attended her a fortnight or so, she asked me "if ever any of my patients felt hungry after my operations?" Being answered in the affirmative, she informed me she was obliged to take a lunch every day I visited her; a thing she had never before done in all her life. She delighted in the application of percussion to the loins, and often insisted on me to apply it with a force which I did not deem admissible. She felt pleasure in it, and as if it positively imparted strength to the weakened parts. The fact was, percussion removed pain, and, when that was effected, the parts performed their functions with ease.

In this case, the grating of the articular surfaces was greatly diminished at every operation, by the passive motion given to the joint. It continued to recur, however, for some time, then became imperceptibly more obscure, and finally ceased altogether. It might be imagined that the friction occasioned by passive motion of the joint, in a dry state, would have induced inflammation in its delicate membranes; but the reverse was the case. Those organs and vessels, whose function it is to prepare a liquor for the lubrication of the joint, were stimulated to a healthy action.

Case XXV.—A lady, about sixty years of age, unmarried, applied to me on the 21st February, 1822, for rheumatism, as she called it, in her neck, shoulders, back, breast, loins, glutei muscles, and hip-joints, accompanied with great debility. These complaints might have been allied to rheumatism in some degree, but that they depended chiefly on a scrofulous diathesis, was evident from many circumstances. One shoulder was higher than the other; the spine was distorted in several places; there was scarcely a rib that retained its original shape: those parts which were of a cartilaginous structure were most affected,—such as the spaces between the vertebræ, the cartilaginous ends of the ribs, the point of the breast-bone, the top of the haunch-bone, the anterior margins of the octabula. The disease had existed a great many years; and the patient's strength was so exhausted, that a very slight exertion, in the way of exercise in the open air, knocked her up completely. The restoration of strength, therefore, was with her the great desideratum; for, except when the parts were handled, or that she attempted exercise, her pains were very bearable. Indeed, she was not aware of the twentieth part of her ailments, till I pointed them out to her: but, having done so, I had no difficulty in persuading her (for she was a sensible woman,) that, in proportion as the parts affected were restored to a healthy action, she would be enabled to take exercise in the open air, and the debility of which she complained would disappear.

Besides the local affections and general debility, there was in this lady's case a mobility, or susceptibility of impression from external objects, which required the utmost delicacy of management. The slightest movement, of which she was not aware, put her all in a tremor. It may well be supposed, then, that the operation of compression and percussion was performed, at first, with the utmost gentleness. But, such were its beneficial effects, that in a short time she could bear it in much greater force; and, in six weeks from the commencement of the operations, pain was entirely removed from all the parts affected with it. As to curing the deformity, it was out of the question: the patient was too far advanced in life, and the parts had been too long consolidated, to warrant the attempt. But she was satisfied with the degree of improvement she had obtained: pain was removed, the nervous system invigorated, so that she was enabled to take exercise in the open air, which restored and confirmed her health. She has enjoyed uninterrupted good health, indeed, ever since my attendance on her, and takes her amusement daily, as if she had never complained. This lady also expressed great delight in percussion; she has it performed every morning by her maid, and at any time of the day when her spirits fag, or when she catches cold. She considers it an invaluable acquisition, and is determined to practise it all the days of her life, as the very best means of preserving health.

CASE XXVI.—I was called, on the 3d of February, 1822, to a woman, aged thirty-five, who had suffered greatly for six weeks from rheumatism in the course of the left collar-bone, at its connexion with the breast-bone and shoulder-blade, in the front of the shoulder-joint, and in the course of the brachial nerve to the bend of the arm. There was also a degree of fever present. The parts affected were always very painful; but, when a shower of snow fell, which was often the case about this time, the pain increased to such a pitch as almost to render the patient frantic. She had not slept an hour at a time for two weeks. Whenever she became warm in bed, the pain was so exasperated that she was obliged to rise out of bed altogether, or to lie above the bed-clothes; a situation I found her in at my first visit. Medicine had a fair trial in this case, without producing any good effect.

In this case, the whole nervous system was greatly affected, precisely like that of a person who has suffered long and severely from toothache; and the parts affected were so exquisitely painful, that they could not be touched but in the most gentle and cautious manner. Percussion directly on the parts was inadmissible; but I applied it at a distance, so as to produce gentle concussion in them, with marked good effect. I proceeded in this manner a week, gradually increasing the force of compression, and slowly approaching the parts themselves in applying percussion, when I had the happiness of being told that my patient could lie a-bed, and enjoy sound sleep. The cure was completed in another week. I think it impossible to conceive a more decisive proof of the efficacy of any remedy, than is here afforded of the benign effects of compression and percussion on the nervous system in a state of morbid irritability.

Case XXVII.—Miss A. E., aged twenty-three, became my patient on the 15th March, 1822, for acute rheumatism. She was constitutionally subject to catarrhal affections; had complained for some time of aching pains in different parts of her body, but had taken to bed a day or two only before I was called. Pulse 120, full and strong; skin hot and dry; considerable thirst; bowels slow. Complained of great pain and stiffness in the shoulders, elbows, wrists, loins, hip-joints, thighs, knees, and ankles. From neck to heel she was quite rigid, and lay in bed as immovable as a piece of cast-iron. I bled her to sixteen ounces, and ordered an aperient medicine; after which, she was to commence taking antimonial wine every two hours, in small doses at first, to be gradually increased as far as the stomach could easily bear. I then applied bandages to the elbows, wrists, knees, and ankles, which proved soothing to the feelings of the patient.

16th.—The bowels have been properly relieved; pulse softer, but not less frequent; thirst not quite so great. Has taken several small doses of the antimonial, and the skin is not so dry; rigidity of the muscles, and local pain undiminished.—Continue the antimonial as directed; the aperient (Epsom salts) as occasion requires; and the bandages.

I now commenced the application of compression and percussion to all the affected parts, giving passive motion at intervals to the joints. These operations were conducted at first with so much gentleness, deliberation, and perseverance, that it required an hour to go through them. As pain receded, however, which it did gradually, the parts could be handled with more freedom; and less time, in proportion, was requisite in going over them. After experiencing their good effects, my patient delighted in the operations, and was daily anxious for the arrival of the hour at which they were to be performed. By removing rigidity and pain from the muscles and joints, they soothed her feelings, and enabled her to turn and move herself in bed; and, so far from exciting the action of the system, the pulse was lowered, both in frequency and force, by every operation. It was uniformly rendered softer, and brought down, sometimes six, at others ten, and at others fourteen, strokes in a minute. By these means, a complete and permanent cure was effected in a fortnight, with very little diminution of the patient's strength.

That compression and percussion resolve local inflammation, and lower the action of the heart and arteries when in a state of morbid excitement, is a statement which, I have no doubt, will excite the surprise of many. It is a matter of fact, however; and facts laugh at scepticism. Besides, the problem, I think, is of easy solution. What, then, is the proximate cause, the essential nature of inflammation? Is it not tension, pain, heat, and redness of a part, occasioned by a preternatural afflux of blood to it? But compression relieves the gorged vessels of an inflamed part, and preserves them pervious; while percussion, by promoting an equal distribution of the blood, precludes its accumulation in a particular part. The local cause of disturbance in the system is, therefore, removed, and the chain of mutual influence between the local and general excitement is broken. Compression and percussion and blood-letting are medicinal powers, equally mechanical in their application and in their proximate effects. They differ, however, in respect, that blood-letting cures by effusion, compression and percussion by diffusion, of the blood. The former debilitates in proportion to the extent to which it is carried; the latter occasions no shock to the system,—subverts none of the balances subsisting between either its proximate or ultimate principles.

Some of my professional readers may say—"Why did he not take thirty or forty ounces of blood at once, and as much more the next or second day, if necessary? By doing so, he would have extinguished fever at once; and the patient would have recovered strength at her leisure." But I would reply, that my object was to cure my patient, not to kill her; to remove disease, without occasioning a shock to the system which (the patient being of a slender make, delicate, and subject to catarrhal affections,) it might never have recovered. I acknowledge, moreover, that I am one of those who prefer efficacious, but safe, remedies, to dashing expedients, which, if they do not cure at once, are sure either to kill or to entail irremediable disease. Besides, copious bleeding, though it often lowers the action of the system with a vengeance, generally leaves rheumatism more firmly fixed than it found it. I have met with many such cases, and cured them by compression and percussion, without the aid of any other power. One gentleman applied to me, after having lost, under the direction of the late Dr. Gregory and Dr. Kelly, of Leith, forty-five cups of blood, which may be averaged at 225 ounces. The patient could crawl, but was incapable of putting on or off his clothes. Two operations of compression and percussion, on two successive days, restored the power of his arms so far as to render him comparatively independent; and the patient himself, assisted by his servant, completed the cure, by following my directions.

Case XXVIII.—On the 16th April, 1822, I was called to Mrs. C., whom I found in a state of great emaciation and debility; pulse 100, with exacerbations in the evening. This lady was evidently of a phthisical constitution, and several of her family had died of consumption; however, she did not labour under any pulmonary complaint at the time. I was called on account of excruciating pain in her right side, hip, thigh, and leg, of eighteen months' standing, and which had rendered her quite lame. The limb was so weak and pained as to be unable to support her weight, which was not great, and she could not move without help and support. On the whole, it is almost impossible to conceive a person in a more delicate, helpless, and distressed condition. She had no expectation I could do her any good; and sent for me merely in compliance with the unceasing solicitations of her friends, who summoned so many undeniable facts to their aid that she found her post untenable, and therefore, as every thing else had failed, consented to make trial of compression and percussion. So completely unhinged was the nervous system, and so tender and painful were the affected parts, that she trembled at the very idea of being touched.

I commenced my operations with a gentleness and caution which a new-born babe could scarcely have felt. I knew, from ample experience, that I should soon soothe the patient's feelings, and dispel her fears. Accordingly, at the end of a quarter of an hour, she could bear to be handled with a freedom which, had it been employed at first, would have demolished her at once. At every touch, I made the nervous power thrill from the top of the haunch to the ankle, with a mixed sensation of pleasure and pain. From this time forth the operations, instead of being dreaded, were courted. Every day my visit was expected with impatience; and, at the end of a week, the pulse was brought down from 100 to 80 in a minute. In a fortnight from the time I was called, pain was entirely removed, the patient could walk perfectly, and has continued to do so ever since. This lady frankly, and *sua sponte*, attributed the melioration of her condition exclusively to my operations, and was pleased to speak of them afterwards with a partiality correspondent to the dread they had formerly inspired.

Case XXIX.—The treatment of the hip-disease has received less improvement from the moderns than, perhaps, any other to which mankind are liable. The practice followed at this very day and hour, is the same in substance with that laid down 2300 years ago. Not but that much valuable information has been obtained respecting the pathology of the disease, and the morbid anatomy of the parts. Nevertheless, little or nothing efficient has been added to the means of cure; the disease remains as intractable, as frightful, and as fatal as ever.

The mode of treatment pursued in the two following cases is not only different from, but apparently incompatible with, that recommended by the best and latest writers on the subject: but it is not to be condemned on that account. Facts will remain facts, in spite of preconceived opinions, and all the ridicule that can be levelled against them. It is no uncommon occurrence, however, to see facts brought against facts, by men of equal reputation and integrity; and yet there may be no paradox in the matter. A difference of circumstances in the application of any remedy, must surely affect the result. If, for instance, a practitioner, after reading this paper, were to adopt my practice, and without discrimination to apply it to a case of hip-disease already advanced to the second stage, he might greatly injure his patient. Were he hence to conclude that the practice is hurtful, instead of being beneficial in such cases, his inference would be perfectly correct; at the same time it would be a complete, though unintentional, misrepresentation of my practice. Nay, were the same practitioner to employ compression and percussion in incipient cases of hip-disease without success, any conclusion he might form against the practice would be erroneous, unless he performed the operation with the tact and skill which, from extensive experience and unremitting attention to the subject for a course of years, I have acquired. I am warranted in making this observation, from the consideration that I daily receive patients, and cure them in a short time, who have laboured for years under rheumatic complaints, and have been treated in every possible way by practitioners of the first eminence, without any good effect. In many such cases, compression and percussion had been resorted to after medicine failed, but with equally little benefit. Compression and percussion have therefore, most erroneously, been pronounced useless, if not hurtful. If the practice was either useless or hurtful, it would succeed in no man's hands; but it succeeds in mine, after it has failed with other practitioners: *ergo*, my mode of applying it must be superior to theirs; and they have no right to deny a practice which is new to them, and success in the application of which can be attained only from experience.

J. R., aged eighteen, became my patient on the 14th of January, 1822, for the hip-disease, right side, of five years' standing. When about thirteen years of age, he one day suddenly experienced a sense of weakness, pain, and stiffness in the joint; to which he paid very little attention, as it soon went off. The same thing continued to recur, however, at irregular intervals, and with various degrees of intensity and duration. At length the complaint returned oftener, and with marked increase of all the symptoms. At the end of three years, during which the disease had been making insidious and imperceptible progress, the patient found his limb unable to sustain his weight, and the stiffness permanent. He could not now throw his leg across a horse. At this stage he had recourse to medical aid, and six wounds were made by escharotics round the joint, and kept open for some time, without affording the smallest benefit. In harvest, 1821, all the symptoms, particularly the pain, became greatly aggravated, and he was henceforth laid up. At this time another surgeon was called, who applied leeches to the joint, and afterwards blisters to the knee. Two large issues were made, by nitrate of silver, in the neighbourhood of the joint at the same time, and kept open for four months, without the least advantage. Mercurial ointment was now rubbed into the thigh, by advice of a third surgeon; which very nearly finished the patient.

When I first saw this young man, which was on the 14th January, 1822, his case was deplorable indeed. The hip and thigh were greatly emaciated, the former appearing quite flat. The skin of the thigh was dry, rough, rigid, and felt like a board. All the glands in the neighbourhood of the joint were swollen, and excruciatingly painful to the touch. Considerable effusion had taken place about the front of the joint, and there was a large fluctuating tumor upon and behind the trochanter minor, but which was entirely free from pain. A perpetual agonizing pain pervaded the whole joint, but was particularly severe on the front, exterior to the course of the femoral artery, reaching down to and behind the trochanter major, along the vastus externus muscle, and terminating in the knee. So intolerable and unremitting was it, that, for eighteen weeks, the patient had been incapable of suffering the limb to remain in any position for an hour at a time, and was therefore, in a great measure, deprived of natural rest. The anterior portion of the crest of the ilium was turned a little outwards, which increased the external cavity of that bone, and clearly evinced the nature of the disease, had there been any doubt on the subject. Pulse 90, small, and irregular; appetite impaired; bowels more regular than could have been expected. The limb was about an inch shorter than the other, and was always kept in a slightly bent position, so that the toes only touched the ground in standing.

I began my treatment of this case by the application of compression and percussion, followed by passive motion of the joint to the extent the patient could suffer it. My expectations of success were any thing but sanguine. I anticipated nothing but suppuration, hectic, and death, —more especially as some members of the family had died of consumption. Notwithstanding, for reasons hereafter to be rendered, I considered this mode of cure the only chance in reserve for my distressed patient. The handling of the parts was at first necessarily very gentle; but I never visited this patient without leaving him easier, in regard to pain, than I found him. On account of the distance at which the patient resided, I saw him every other day only: consequently, a much longer time elapsed before any decided impression was made, than would have been requisite had the operations been performed daily. At the end of three weeks, however, his condition was so much ameliorated that he could sleep nine hours without interruption. This was of immense advantage, not only to the patient himself, but to his friends, one or other of whom had to attend him every now and then, even during the night, to alter the position of the limb. But, though the effects of the operations were now more obvious, decided, and permanent, the parts affected remained extremely tender to the touch, and unaltered in appearance. I spent an hour, at least, with the patient at every visit. One day I operated an hour and a half on the flexure of the joint and margin of the acetabulum, with the view of getting at some defined spots, which were extremely painful, and impeded the motion of the joint. I accomplished my object; and, though the patient was much exhausted, not only no bad consequences resulted from the exertion, but he never was so much pained afterwards. I now proceeded, therefore, with more boldness and effect.

The patient's general health, and consequently the diseased parts, were greatly affected by the state of the weather. The process of amendment was often suspended, and sometimes the symptoms retrograded, or threatened to do so. On such occasions I redoubled my diligence, and always with the desired effect. The operation of compression and percussion never failed to exert a benign and sanative influence on the parts affected, and also on the whole nervous system. This was not a vague conjecture; it was an effect, of which the senses were the evidence. The patient knew when he was relieved from pain; and I knew when he could suffer the parts to be handled with increased freedom, and when the motion of the joint was facilitated.

The reader may startle, perhaps, at the idea of compression and percussion changing the nervous system, or any part of it, from a morbid to a healthy state or action. It is, nevertheless, true. I have observed it in a thousand instances. In the present case, when I grasped the anterior muscles of the thigh below the trochanters, and raised them from the bone, the patient roared out from pain: when I compressed them, his sensations were pleasurable. Compression and percussion completely changed this morbid state of the muscles, so that they could be handled, and raised, and twisted at pleasure. Is this not changing the morbid state of the nerves into a healthy? The very skin was changed from a dry and rigid to a naturally moist and pliant state. The patient himself made the observation first. I have in many instances observed the same effect produced by compression and percussion; and the fact admits of an easy explanation. When emaciation of the muscles takes place, the circulation in the capillary vessels must be languid in proportion: the latter, indeed, must be the immediate cause of the former; for, if the ultimate arteries continued to perform their functions equally all over the body, no emaciation of any particular part could take place. When, therefore, compression and percussion are applied to parts in a state of emaciation, the ultimate arteries are roused to action, the blood is brought to the surface and circulated equably;* the nervous power is conciliated and diffused; and, consequently, all the functions dependent on these must be performed more perfectly.

About the month of May, the patient complained of pain on the right side of the lumbar vertebræ, along the crest of the ilium, and in the muscles connected with these parts. He began to lean very considerably to the side affected, which gave the appearance of a curvature to the spine. When these parts were handled, the pain was most excruciating, which made me suspect it was all over with my patient, as one part was no sooner relieved than another was attacked with disease. However discouraging these circumstances, I continued my operations, and succeeded in removing the pain from the loins, much sooner than I expected; and it has never returned. The spine is also perfectly straight. But, though pain and effusion were removed, in a great degree, in a few weeks after the commencement of the operations, yet several months elapsed before any increase of flesh could be perceived. This might be owing as well to the wavering and imperfect state of the patient's general health, as to the local disease. About the month of June, however, his general health became more steady and confirmed, and an increase of flesh over the whole body, not excepting even the diseased limb, was very manifest. Finally, at this date, 12th November, 1822, the patient is in perfect health; pulse natural (66); the joint and limb are, and have been for a considerable time back, permanently free from pain; the tumor at the head and inside of the thigh is decreased one-third, is flatter, more moveable, and more compressible; effusion in other parts round the joint has disappeared; the glands are reduced to their natural size; rotatory motion is very considerable, not perfect; flexion and extension are perfect; the patient can rise from his back, with both limbs extended on a plane; standing erect, he can plant the foot of the weak limb on a chair; he can touch his chin with the knee, and his hip with his heel, and can place his heel on the opposite groin. The limb is gaining flesh daily; the margin of the gluteus maximus is beginning to assume its natural prominence; and the patient can walk without crutch or support of any kind. I saw his mother twelve months afterwards, who informed me, his health was completely con-

* This was beautifully illustrated in the present case, from the circumstance that, before I was called, the issue behind the trochanter major had been healed up for some time, but the scar retained an uncommonly livid colour. Compression and percussion had not been long applied, however, when it changed to a more healthy red, and gradually acquired a natural appearance. I made no mention of the change till it was noticed by the patient's mother, who observed that, "surely the operations were doing good, for the very colour of the wound looked better."

firmed, the tumor had nearly disappeared, and that the limb was perfectly serviceable.

I took several of my professional friends to see this case, who agreed that it was unique; and I think I may add that, if ever premises warranted a conclusion, the history of this case authorises me to say, had the patient been treated by compression and percussion at the commencement of his illness, or even after it had existed for some years, the disease would have been arrested *in limine*, and all the subsequent distress which he suffered, would have been prevented.

In addition to the facts of the case, it will naturally be expected I should assign my reasons for attempting to cure by *motion* a disease, for which *perfect rest* is universally prescribed. They are as follow :—

The success I have had for a number of years in curing rheumatism, of every degree of inveteracy, by compression and percussion, has induced many to apply to me for the cure of chronic pains arising from ricketty and scrofulous constitutions, the patients imagining their complaints to be merely rheumatic. Whoever, indeed, has had much experience in the treatment of rheumatism, must have met with many such cases. I always consider that case to originate in scrofula, where cartilaginous parts and their coverings are chiefly affected, and where there is general debility at the same time. For instance, when the pain is confined to, or chiefly affects, the cartilages of the ribs, breast-bone, vertebræ, crest of the ilium, or the joints, attended with general debility, I consider the disease to proceed from a scrofulous constitution. Now, in applying compression and percussion to the soft or muscular parts, which in such cases are also often very painful, and sometimes knotted, I found the practice attended with the most beneficial effects on the cartilaginous parts also; and that it removed that undefinable exhausting pain and uneasiness, which are both a cause and an effect of the general debility accompanying such complaints. I therefore came to follow the same practice in every such case as I did in cases of pure rheumatism, and with equal success. Compression and percussion, by removing pain, indirectly strengthens the patient; more exercise can be taken in the open air ; the digestive organs are improved, and the general health is restored. Having, therefore, observed such effects from compression and percussion in cases of chronic pains and general debility, arising from a scrofulous taint in the constitution, it was most natural for me to conclude that the same practice would be beneficial in scrofulous affections of the hip-joint. Thus, the adoption of the practice followed in this most interesting case, was not a random experiment—a bow at a venture, but the result of observation and induction.

Pathologists agree in attributing scrofula and rickets to a deficiency of phosphate of lime, as their proximate cause, arising either from an inordinate action of the absorbents or from deficiency of action in the ultimate arteries, whose function it is to secrete phosphate of lime. From the effects of compression and percussion, as illustrated in the preceding case, I apprehend the latter to be the truth; for, under their application, the progress of debility is arrested, the distortion of bones is rectified, and the bones themselves are restored to their former strength. These effects must be owing to the renewed secretion of phosphate of lime, produced by the excitement of the ultimate arteries: and, if compression and percussion promote the secretion of phosphate of lime in the bones, much more so in the soft and fluid parts; for it is a proximate principle of the soft and fluid parts of the body, as well as of the bones. It is, therefore, easy to comprehend, that a mode of treatment which promotes the renewal of a proximate principle, in the deficiency of which debility consists, should arrest the progress of debility.

Case XXX.—I was called to Mr. S., aged sixty-six, on the 20th April, 1822. About fourteen weeks before this, he fell one evening, going down a stair, on his right haunch, and felt so much hurt, that he could not return home without help. A surgeon was immediately called, who declared that neither fracture nor dislocation had taken place; and, of course, that the accident was of no consequence. The patient continued for some days, however, to experience considerable pain in the limb, from the hip-joint to the ankle inclusive, which prevented motion entirely. Another surgeon was now called in, who also pronounced the injured parts to be free from fracture or dislocation, and recommended that they should be anointed with linimentum ammoniatum. This gentleman must also have considered the case as of no importance, as he did nothing farther in it. Notwithstanding, the parts around the hip-joint continued painful to the touch, and therefore were never touched. Any attempt at motion of the joint was also attended with excruciating pain; of course, the limb was constantly kept in a state of perfect rest. The patient lay in bed for the most part, or, if he came out of it, he made use of a crutch, and kept the limb in the half-bent position; so that the joint suffered no more motion than if it had been a continuous bone. Fourteen weeks passed over in this way, without the smallest prospect of his ever being able again to set his foot to the ground; though there was no where any external appearance of injury or disease: the whole limb was pained, and so œdematous below the knee, that the skin was about to crack in many places.

Had the patient's constitution been sound, the accident that befel him would most likely have had no bad consequences. He was a light-made man, and, as has been observed, there was no external appearance of injury, even on the parts which came in contact with the stones on which he fell; so that the gentlemen who preceded me were perfectly justified in considering the case as trifling. But, after the lapse of so many weeks, during which any taint that might exist in the constitution had time to develop itself, matters assumed a different aspect. There could now be no manner of doubt that the case was one of hip-disease, occasioned by the accident. The pain in the hip-joint and at the knee, on attempting motion,—the grating of the articular surfaces, on passive motion being given to the joint,—the aspect of the patient,—all concurred in proving that the effects of the accident proceeded from a scrofulous taint in the constitution, brought into activity by external violence.

Expecting I would satisfy myself with prescribing some liniment to the parts, the patient, when I began my operations, regarded me with astonishment, and hesitated not to affirm that, instead of being benefited, much injury could not fail to result from such a mode of proceeding. Indeed, had it not been for the respect I entertained for his friends, I would not have touched this obstinate and unreasonable man a second time. After a few operations, however, when he found the motion of the joint increasing, and the pain in and about it decreasing, the reception I met with was very different. Now he was all deference, submission, and apology. The consequence was, that in a week the motion of the hip-joint was restored; and, in three weeks from the time I was called in, he was walking the streets. The œdema of the leg had by this time also disappeared.

Here, then, is another case of hip-disease, the progress of which was arrested by compression and percussion. It may be said that other means might have arrested it as well as compression and percussion, had they been tried. I do not deny it. I only affirm, that compression and percussion, combined with passive motion of the joint, did arrest the disease, and in a much shorter time, too, and with much less injury to the system; because a copious and long-continued discharge of purulent matter, by issues or otherwise, though it may sometimes cure the local affection, much oftener saps the foundation of the constitution. It cannot, in the nature of things, be otherwise. Was there ever yet a human being seen to have a healthy appearance, with an active issue of any continuance? Compression and Percussion, on the contrary, strengthen the whole frame: consequently, the patient is much sooner enabled to avail himself of exercise in the open air, which, as it is necessary to the preservation of health, must contribute essentially to the cure of disease. Had I, in the case under consideration, advised the repeated application of leeches, of scarification and cupping, and ultimately issues in the neighbourhood of the joint,—what would have been the inevitable consequences? Much longer confinement and great diminution of strength, which, combined, might have given a fatal predominance to the scrofulous diathesis.

From the effects of compression and percussion, combined with passive motion of the joint, in this case, after matters had assumed so formidable an aspect, it certainly is not too much to infer that, had these means been employed at the beginning, the consequences of the accident would not have occurred. It is much easier, surely, to prevent than to cure disease; and that remedy which has the power of arresting the progress of any malady after it is confirmed, would unquestionably prevent its proceeding to any extent, if early employed. This is as clear and conclusive as that twice two make four. How many, then, of both sexes, and in all ranks of life, might be rescued from an early grave, or from irremediable debility and deformity, by timely combining with other remedies the simple operation of compression and percussion!

[To be continued.]

